# Substrate degradation and black soldier fly larvae bioconversion performance profile on co-digested oil palm biomass-based feedstock

**DOI:** 10.1371/journal.pone.0332046

**Published:** 2025-09-15

**Authors:** Mulki Salendra Kusumah, Frisda Rimbun Panjaitan, Bagus Giri Yudanto, Brahmani Dewa Bajra

**Affiliations:** Indonesian Oil Palm Research Institute, Medan, North Sumatra, Indonesia; Bina Nusantara University, INDONESIA

## Abstract

Utilization of oil palm empty fruit bunches (OPEFB) is limited due to its low nutrient value but high lignocellulose content. Black soldier fly larvae (BSFL, *Hermetia illucens* L.) is capable of converting organic materials into high-value products. A co-digestion strategy combining low-nutrition OPEFB and other high-nutrient oil palm by-products, i.e., oil palm kernel meal (OPKM), is an approach to enhance the digestibility of oil palm biomass by BSFL. However, the information on the degradation of cellulose, hemicellulose, and lignin in OPKM, OPEFB, and their co-digested substrates after treatment with BSFL is scarce. Therefore, this experiment evaluated the growth and bioconversion potential of BSFL grown on OPKM, OPEFB, and their mixture. Three feed treatments of 100% OPEFB, 100% OPKM, and a mixture (50% OPEFB: 50% OPKM) were given to larvae to observe growth, bioconversion potential of BSFL, and substrate degradation. In addition, the changes of the substrates morphology were analyzed by scanning electron microscope (SEM). The results show that an increase of larval biomass weight by 634% was obtained in a substrates combination of OPEFB and OPKM, with a reduction in the feed conversion ratio to 84% in twenty-five days, as compared to a 100% OPEFB feed. SEM results indicate that the surface of OPKM and OPEFB altered significantly after being consumed by BSFL. Ultimately, the current findings confirmed the potential of BSFL as bioconversion agent in the utilization of low-nutrition organic materials with high lignocellulosic content, such as OPEFB by using co-digestion method. This finding is promising to be implemented for treatment and utilization of palm oil mills by-products.

## Introduction

Global demand of palm oil has been ever increasing, thus leading to oil palm plantation expansion. From 2000 to 2021, harvested oil palm area in Indonesia increased by 650%, while Malaysia recorded a 50% increase. The rapid expansion has resulted in a significant increase in palm oil production, with Indonesia’s output increasing by 566% and Malaysia’s by 65%s [1]. As production increases, so does the amount of waste and by-products, including oil palm empty fruit bunches (OPEFB) and oil palm kernel meal (OPKM). These by-products are estimated to considerably contribute to agricultural waste, with OPEFB alone generating 230 kg for every ton of processed fresh fruit bunches [2]. Consisting of cellulose (25.63–44.23%), hemicellulose (0.01–42.49%), and lignin (3.7–47.1%), OPEFB is considered as an oil palm by-product that is rich in lignocellulose yet low in nutrients [3]. OPEFB takes six months for natural decomposition and it is indigestible by most organisms due to its high lignocellulose content [4]. The accumulation of OPEFB might led to environmental issues such as the spread of pathogenic pests and microorganisms, the requirement for extensive areas for disposal, and gas emissions resulting from its incineration [5].

Black soldier fly larvae (BSFL), *Hermetia illucens* (L.) (Diptera: Stratiomyidae), is a well-recognized non-pest fly species that elaborates gut microbiota to convert various organic materials into highly valued products [6,7]. The polyphagous diet of BSFL shows by its impressive wide substrate preferences diversity. Numerous studies documented the BSFL bioconversion potential of various organic materials and waste, such as oil palm by-products [8], spent coffee grounds and blood meal [9,10], restaurant waste [7], fruit waste and sewage sludge [11], and animal manure [12,13] into high-quality biomass.

The issue concerning OPEFB utilization as feed and growth medium for the BSFL is mainly due to its low nutritional value and high lignocellulose content [4]. The co-digestion method is an approach that combines multiple types of organic waste to balance nutrient composition, improve waste bioconversion, and potentially, optimize BSFL growth [13]. Previous reports have documented the beneficial impacts achieved in BSFL feeding through the co-digestion of organic waste method [9,13]. In an experiment by Navajas-Porras et al. [9], BSFL fed using the co-digestion method with a mixture of blood meal and spent coffee grounds yielded a higher biomass (110.00 ± 10.00 mg) than those fed solely on blood meal (90.00 ± 20.00 mg) or spent coffee grounds (80.00 ± 20.00 mg). Similarly, the BSFL that was fed a co-digestion method that included dairy and chicken manure shows better development time, bioconversion efficiency, and feed conversion ratio values (20.00 ± 0.58 days; 7.45 ± 0.05%; 3.82 ± 0.28, respectively) in comparison to BSFL that was fed only on dairy manure (24.33 ± 1.76 days; 6.99 ± 0.28%; 4.22 ± 0.42, respectively) [13]. Therefore, a co-digestion approach consisting of OPEFB and nutrient-rich OPKM was investigated for the development of BSFL, aiming at utilizing low-nutrition OPEFB.

Currently, no reports have been found on the evaluation of the degradation of cellulose, hemicellulose, and lignin in OPKM, OPEFB, and their co-digested substrates after treatment with BSFL. This experiment aims to assess the growth (survival rate, development time, and larval production) and bioconversion potential (waste reduction, bioconversion efficiency, and feed conversion ratio) performance of BSFL grown on a mixture of OPKM and OPEFB. Another objective of this experiment was to assess the degradation of cellulose, hemicellulose, and lignin by BSFL on OPKM, OPEFB, and the co-digestion mixture substrates. Discovering strategies for OPEFB utilization, such as the production of BSFL biomass, not only mitigates environmental liabilities linked to waste management and disposal but also generates additional revenue for the oil palm industry.

## Materials and methods

### Material

OPEFB and OPKM were obtained from the oil palm industry in Aek Pancur, Deli Serdang, North Sumatra, Indonesia (3.496500, 98.813562). The OPKM was sieved (mesh: ø15 mm). Meanwhile, a shredding (mesh: ø10 mm) and hammermill machine (mesh: ø1 mm) was used to process the OPEFB.

### Colony maintenance

A colony of black soldier flies was established in 2021 at Laboratory of Mechanization, Post-Harvest, and Environmental Conservation in Indonesian Oil Palm Research Institute (IOPRI), Medan, North Sumatra, Indonesia. Prior to being used in this experiment, the IOPRI BSF colony was maintained on oil palm by-products, adhering to protocols stated in Bajra et al. [4]. In order to facilitate BSF breeding, the adult flies have been emerging in a dark chamber that was kept at 20 °C and approximately 60–70% relative humidity [14]. Polyethylene plastic was used to cover a 300 × 200 × 100 cm green nylon breeding cage that was set up in a semi-open space. Meanwhile, the ovitrap was made by a stack of thin wood tied up with a rubber band. The ovitrap were moved to an empty bin for natural hatching after being checked daily for freshly laid eggs. The newly hatched BSFL (neonates) were used as the subject of this experiment.

### Experimental design

Three treatments were conducted: 100% OPEFB, 100% OPKM, and a mixture of 50% OPEFB and 50% OPKM, providing as feed and growth media for BSFL. Three replicate of 2000 neonates were reared in plastic trays (64 × 41 × 15 cm^3^) and fed with different feed treatment at feeding rates of 75 mg/larvae/day [15]. The moisture content of the BSFL feed and experimental growth medium (frass) was maintained at 70 ± 5%. The rearing conditions for the experimental operation were maintained at 25–27 °C and 60–70% relative humidity [14]. Twenty BSFL were randomly picked from each tray as a sample [16]. The samples were weighed using a Shimadzu ATX324R laboratory scale (accuracy: 0.001 g) to determine the fresh larvae weight, followed by microwave drying. Subsequently, samples of feed and frass were collected for the determination of dry matter, water content, protein, lipid, lignin, cellulose, hemicellulose, total organic carbon, total nitrogen, and C/N ratio.

### BSFL growth and bioconversion potential parameters

The fundamental feasibility of utilizing OPKM, OPEFB, and their co-digestion mixtures as BSFL growth and development substrate was assessed by evaluating survival rate, development time, and larval production of BSF. The development time stopped once 5% of the larvae reached the prepupal stage, followed by an assessment of the survival rate, fresh BSFL weight, and BSFL length [17]. The number of neonates at the beginning of the experiment and the number of larvae at the end of the life cycle were used to calculate the larval survival rates [4]. The following formula is used to calculate the survival rate (Equation 1):


$$\text{Survival rate }(\%)=\;\frac{Final\;amount\;of\;larvae\;(n)}{Initial\;amount\;of\;neonates\;(n)}\;\times \text{100}\%$$ (1)

A few important parameters used to assess BSFL as a potential bioconversion technology are waste reduction, bioconversion efficiency, and feed conversion ratio (FCR) of the substrates. The BSFL was separated from the feed, and the waste reduction (Equation 2) and FCR (Equation 3) were calculated following the equations as stated by Broeckx et al. [18], while the bioconversion efficiency (Equation 4) was calculated using the equation stated by Rehman et al. [12]:


$$\text{Waste reduction }(\%)=\frac{\text{Total feed added }(\text{gram},\text{ dry matter})\;-\text{ Residues }(\text{gram},\text{ dry matter})\;}{\text{Total feed added }(\text{gram},\text{ dry matter})}\times \text{100}\%$$ (2)

$$\text{FCR }=\;\frac{\text{Total feed added }(\text{gram},\text{ dry matter})}{\text{Final larval }(\text{gram},\text{ wet matter})\;-\text{ Initial larval }(\text{gram},\text{ wet matter})}$$ (3)

$$\text{Bioconversion efficiency }(\%)\;=\;\frac{\text{Final larval }(\text{gram},\text{ dry matter})\;}{\text{Total feed added }(\text{gram},\text{ dry matter})}\times \text{ 100}\%$$ (4)

### Chemical analysis

The concentration of protein (SNI 01.0008.1987), lipid (MPOB K 1.3–2004), total organic carbon, total nitrogen, and C/N ratio (SNI 01.0008.1987) from the feed were analyzed according to the procedures reported in previous research [4]. The methods described by Zhang et al. [13] were used to evaluate the concentrations of lignin (SNI 14.0492.1990 A), cellulose (SNI 14.0444.1989 A), and hemicellulose (SNI 14.1303.1989 A) in the OPKM, OPEFB, and the mixture substrates.

### Scanning electron microscopy (SEM)

The qualitative study of the morphology changes in the OPKM, OPEFB, and their mixture substrates during digestion with BSFL was conducted using a scanning electron microscope (SEM) (Hitachi model SU3500 SEM, Japan) running at an accelerating voltage of 3.0 kV. Double-sided tape was used to attach the dried samples on aluminum stubs. A thin layer of gold (15 nm for 10 min) was sputtered on the mounted samples to improve their conductivity thereby enhancing the quality of the images.

### Data analysis

The significance of the protein, lipid, lignin, cellulose, hemicellulose, total organic carbon, total nitrogen, C/N ratio, survival rate, development time, fresh BSFL weight, BSFL length, waste reduction, bioconversion efficiency, and FCR were all assessed statistically using a statistical software program (JASP-0.19.3.0, Netherlands). All treatments and measurements were conducted in triplicate. A one-way analysis of variance (ANOVA) was followed by a pairwise comparison test using Tukey’s HSD post hoc to confirm the statistical differences. Significant values were defined as P < 0.05.

## Results and discussion

### Physicochemical characteristics of OPKM, OPEFB, and the mixed substrates

The physicochemical characteristics of the OPKM, OPEFB, and the mixed substrates used in this experiment as a feedstock are presented in Table 1.

**Table 1:** Physicochemical characteristics of the OPKM, OPEFB, and the mixed substrates.

Parameters	OPKM	OPEFB	Mixed substrate
Water Content (%)	7.88 ± 0.02a	4.65 ± 0.02b	6.27 ± 0.02c
Dry Mass (%)	92.12 ± 0.02b	95.35 ± 0.02a	93.73 ± 0.02c
Protein (%)	19.98 ± 0.02a	3.68 ± 0.03c	14.33 ± 0.19b
Lipid (%)	5.17 ± 0.61a	0.39 ± 0.06c	1.85 ± 0.46b
Cellulose (% dry mass)	33.15 ± 0.57a	41.78 ± 0.64b	33.35 ± 1.24a
Hemicellulose (% dry mass)	21.18 ± 0.19b	19.20 ± 0.08c	25.99 ± 0.60a
Lignin (% dry mass)	25.65 ± 0.77a	19.00 ± 0.73c	20.64 ± 0.64b
Total Organic Carbon (% dry mass)	55.10 ± 0.10a	49.69 ± 0.25c	53.49 ± 0.21b
Total Nitrogen (% dry mass)	3.19 ± 0.01a	1.41 ± 0.01c	2.29 ± 0.03b
C/N ratio	17.24 ± 0.02b	35.12 ± 0.43a	23.33 ± 0.33c

*The table presents values as means with standard deviations. Statistical analysis (ANOVA “Tukey’s post-hoc pairwise comparison test”) indicated no significant difference (P > 0.05) between values denoted by the same letter within the same rows.

As shown in Table 1, OPKM has a higher protein, lipid, organic carbon, and nitrogen content compared to OPEFB. The result shows that OPEFB had significantly lower protein and lipid contents (3.68 ± 0.03% and 0.39 ± 0.06%, subsequently), while the other two substrates showed higher values of 19.98 ± 0.02% of protein and 5.17 ± 0.61% of lipid for OPKM, and 14.33 ± 0.19% of protein and 1.85 ± 0.46% of lipid in the mixed substrate. The protein and lipid content of feeds was recognized as the primary factor that promotes BSFL growth and development [19,20].

Lignocellulosic biomass consists of three main polymeric components, namely: cellulose (27–50%), hemicellulose (24–35%), and lignin (18–25%), which are intricately interconnected [21]. Substrates containing OPKM shows lower cellulose content, with values of 33.15 ± 0.57% for the OPKM-only substrate, and 33.35 ± 1.24% for the mixed substrate, compared to the OPEFB substrate which reaches 41.78 ± 0.64%. On the other hand, the mixed substrate shows the highest hemicellulose content amongst other treatments, resulting at 25.99 ± 0.60%, whereas the OPEFB shows the lowest hemicellulose content at only 19.20 ± 0.08%, and the OPKM substrate shows 21.18 ± 0.19% of hemicellulose. The lignin content was higher in OPKM and the mixed substrate compared to OPEFB. The lignin content of OPEFB is 19.00 ± 0.73% whilst OPKM lignin content is 25.65 ± 0.77%. On the other hand, that of the mixed substrates is 20.64 ± 0.64%. In an experiment conducted by Zhang et al. [13], it was found that high lignocellulose material can be a factor that limits the growth and development of BSFL.

The analysis of organic carbon and nitrogen content demonstrated that OPEFB had significantly lower values (49.69 ± 0.25% and 1.41 ± 0.01%, subsequently), compared to other two substrates which shows higher values of 55.10 ± 0.1% of organic carbon and 3.19 ± 0.01% of nitrogen in OPKM, and 53.49 ± 0.21% of organic carbon and 2.29 ± 0.03% of nitrogen in the mixed substrate. Thus, resulting in significantly highest C/N ratio in OPEFB substrate, measured at 35.12 ± 0.43, while OPKM and mixed substrate, shows the C/N ratio of 17.24 ± 0.02 and 23.33 ± 0.33, respectively. Organic carbon contributes as a primary energy source for metabolism and enhances the efficiency of substrate conversion into larval biomass [22]. High nitrogen in the feed can increase protein accretion in BSFL [19]. A feed with optimum C/N ratio and balanced nutrition would enhance the growth and development of BSFL [23].

In this experiment, OPKM considered as an organic material that has a higher nutritional content and should be easily digested by BSFL. On the other hand, OPEFB has hypothetically lower digestibility than OPKM. Thus, mixing these two organic materials as a new feed formulation result in a better variety of nutrients that can be digested and consumed by BSFL compared to OPEFB as a single feed.

### Survival rate, development time, and BSF larval production

Survival rate, development time, and larval production of BSF were evaluated to determine the fundamental feasibility of an organic material as BSFL growth and development medium. The composition of organic matter and nutrients of the growth media significantly affected the growth and development of BSFL [12,19,20]. Survival rate, development time, and biomass production of BSFL reared on OPKM, OPEFB and mixed substrate are shown in Table 2.

**Table 2:** Survival rate, development time, and larval production of BSFL reared on OPKM, OPEFB and the mixed substrate.

Feeding Treatment	Survival Rate (%)	Development time (days)	Fresh Weight		Individual larval length (mm)
			Total Larval (g)	Individual Larval (mg)	
OPKM	86.16 ± 0.02a	20 ± 0.02b	1,191.78 ± 0.37a	203.79 ± 4.27a	21.50 ± 0.5a
OPEFB	66.91 ± 0.15c	40 ± 0.02a	69.40 ± 0.13c	10.70 ± 0.17c	8.66 ± 0.57c
Mixed substrate	77.63 ± 0.19b	25 ± 0.02c	442.19 ± 1.09b	78.50 ± 0.19b	16.95 ± 0.05b

*The table presents values as means with standard deviations. Statistical analysis (ANOVA “Tukey’s post-hoc pairwise comparison test”) indicated no significant difference (P > 0.05) between values denoted by the same letter within the same columns.

The BSFL survival rate was significantly higher in the OPKM-fed group compared to the mixed substrate and OPEFB-fed treatment groups. The BSFL survival rate in OPEFB-fed treatment groups only reached 66.91 ± 0.15%, whilst the OPKM-fed treatment group reached 86.16 ± 0.02% of survival rate. On the other hand, mixed substrate-fed treatment group reached a survival rate of 77.63 ± 0.19%. The BSFL development time in this experiment refers to the initial transformation of larvae into prepupae within the colony. The development time was 20 days for BSFL fed with OPKM and 25 days for the mixed substrate-fed treatment group, whilst OPEFB-fed BSFL treatment group exceed 40 days to develop. OPKM-fed BSFL treatment group shows significant increase in the biomass weight and length (203.79 ± 4.27 mg and 21.50 ± 0.5 mm, respectively), whereas in the mixed substrate treatment groups, it only reaches 78.50 ± 0.19 mg in term of individual larval weight and 16.95 ± 0.05 mm in term of length. BSFL fed with OPEFB shows lowest growth, as they weigh only 10.70 ± 0.17 mg and grow only up to 8.66 ± 0.57 mm.

In this experiment, the co-digestion method enhanced the quality of OPEFB, thereby positively improving the growth and development of BSFL. Diets containing important macronutrients, including proteins, lipids, and carbohydrates, offer the energy necessary for the BSFL to accumulate body biomass and shorten development time [19,20]. OPKM as the more digestible and nutritious feedstock helps to accelerate the development time, also increases the survival rate and larval production. The higher protein content in OPKM possibly contributed significantly to larval growth, as protein is essential for the synthesis of larval tissues and overall development [24]. This discovery aligns with the experiment of Albalawneh et al. [20], which discovered that BSFL grown on organic substrates high in proteins and lipids (e.g., chicken feed) revealed better growth performance (201.3 mg/larva on day 20) compared to those grown on less nutritious sludge substrates (between 51.2 and 162.7 mg/larva on day 20).

The result is in line with the experiment from the co-digestion method conducted by Bajra et al. [4], that BSFL fed with OPKM and fine OPEFB achieved a maximum survival rate of 96.67 ± 7.57% in the BSFL group with a feed composition of 80% PKM and 20% fine OPEFB. The incorporation of reduced PKM in the feed combination led to reduced survival rates in comparison to the mixture with higher PKM concentration. This demonstrates the preference of BSFL for OPKM as the most easy-to-digest raw material in the mixture, in contrast to OPEFB. Similar studies are conducted on co-digestion of dairy manure and chicken manure were observed that enhanced the growth and developmental performance of BSFL [12,13].

### Waste reduction, bioconversion efficiency, and feed conversion ratio of BSFL

The result of waste reduction, bioconversion efficiency, and feed conversion ratio (FCR) parameters in this experiment was shown on Table 3.

**Table 3:** Waste reduction, bioconversion efficiency, and feed conversion ratio of BSFL converting OPKM, OPEFB and the mixed substrate.

Feeding Treatment	Waste Reduction (%)	Bioconversion Efficiency (%)	Feed Conversion Ratio
OPKM	80.34 ± 0.86a	16.81 ± 0.01a	2.13 ± 0.01a
OPEFB	56.35 ± 1.17b	1.03 ± 0.01b	36.72 ± 0.07c
Mixed substrate	76.57 ± 0.70a	5.18 ± 0.01a	5.74 ± 0.01b

*The table presents values as means with standard deviations. Statistical analysis (ANOVA “Tukey’s post-hoc pairwise comparison test”) indicated no significant difference (P > 0.05) between values denoted by the same letter.

The lowest percentage of waste reduction was observed in OPEFB treatment (56.35 ± 1.17%) compared to other treatments. The treatment that contained OPKM revealed a higher percentage of waste reduction, 80.34 ± 0.86% (OPKM only) and 76.57 ± 0.70% (mixed substrate), although there were no significant differences. The highest bioconversion efficiency was observed in treatments with OPKM, either as a sole substrate or as a mixed substrate, resulting in 16.81 ± 0.01% for OPKM-only treatment groups and 5.18 ± 0.01% for mixed substrate treatment, whereas the treatment with OPEFB as a single substrate exhibited an efficiency of only 1.03 ± 0.01%. Similarly, the FCR analysis findings indicated that the BSFL treatment with OPKM yielded significantly lower FCR values of 2.13 ± 0.01 (solely OPKM-fed) and 5.74 ± 0.01 (mixed substrate-fed), compared to the OPEFB treatments, which revealed the highest value (36.72 ± 0.07).

The waste reduction, bioconversion efficiency, and FCR parameters are crucial to evaluate BSF larvae potential to convert a biomass [18]. The mixed substrate treatment group had an average waste reduction of 76.57 ± 0.70%. This aligns with the waste reduction observed in earlier studies utilizing OPKM and OPEFB, which reported a waste reduction of 71–72% [4]. This finding is higher when compared with studies of chicken feed, a staple control feed in BSF growth experiment (54.10%) [20], and co-digestion of dairy manure and chicken manure experiment (44.32 to 53.38%) [12]

The addition of OPKM in co-digestion mixtures enhances substrate digestibility and nutrients for BSFL, thereby increasing the mean bioconversion efficiency value. High nutrients substrates not only facilitate larval growth but also enhance bioconversion rates by enabling larvae to convert waste into valuable biomass more effectively [9]. Lack of nutrients substrate as a feedstock would stunt BSFL growth due to insufficient macronutrients, resulting in delayed development and decreased waste-processing efficiency [20]. This experiment demonstrated a 502.91% increase in bioconversion efficiency upon the addition of OPKM to the OPEFB substrate compared to OPEFB-only substrate treatment groups. A similar bioconversion efficiency improvement was observed in the co-digestion method conducted by Rehman et al. [12] with dairy manure and chicken manure, increasing from 4.19% (solely dairy manure) to 5.88% and 7.90%. Optimizing the nutritional composition of substrates can improve the ecological and economic advantages of BSFL-based waste management systems, resulting in increased yields of protein-rich biomass for animal feed applications [20].

A lower FCR value implies better conversion efficiency, as it indicates higher net larval production from an equivalent feed supply [18]. The decreased FCR value in the co-digestion treatment demonstrated that BSFL effectively adapted to the improvements in feed nutritional quality, yielding increased biomass production from the provided feed amount. The high protein content in OPKM (19.98 ± 0.02%) is one of the main factors in reducing the FCR values. Larval growth potential is also stunted if protein digestibility is poor [18]. Previous study using apple pulp and chicory roots reveals insufficient protein content resulted in suboptimal larval growth [18]. These findings highlight the application of co-digestion method for waste management using BSFL, that can improve sustainable bioconversion processes and optimize nutrient recycling from agro-industrial by-products, especially from oil palm industry.

### Composition changes of the substrates

**Fig 1:**
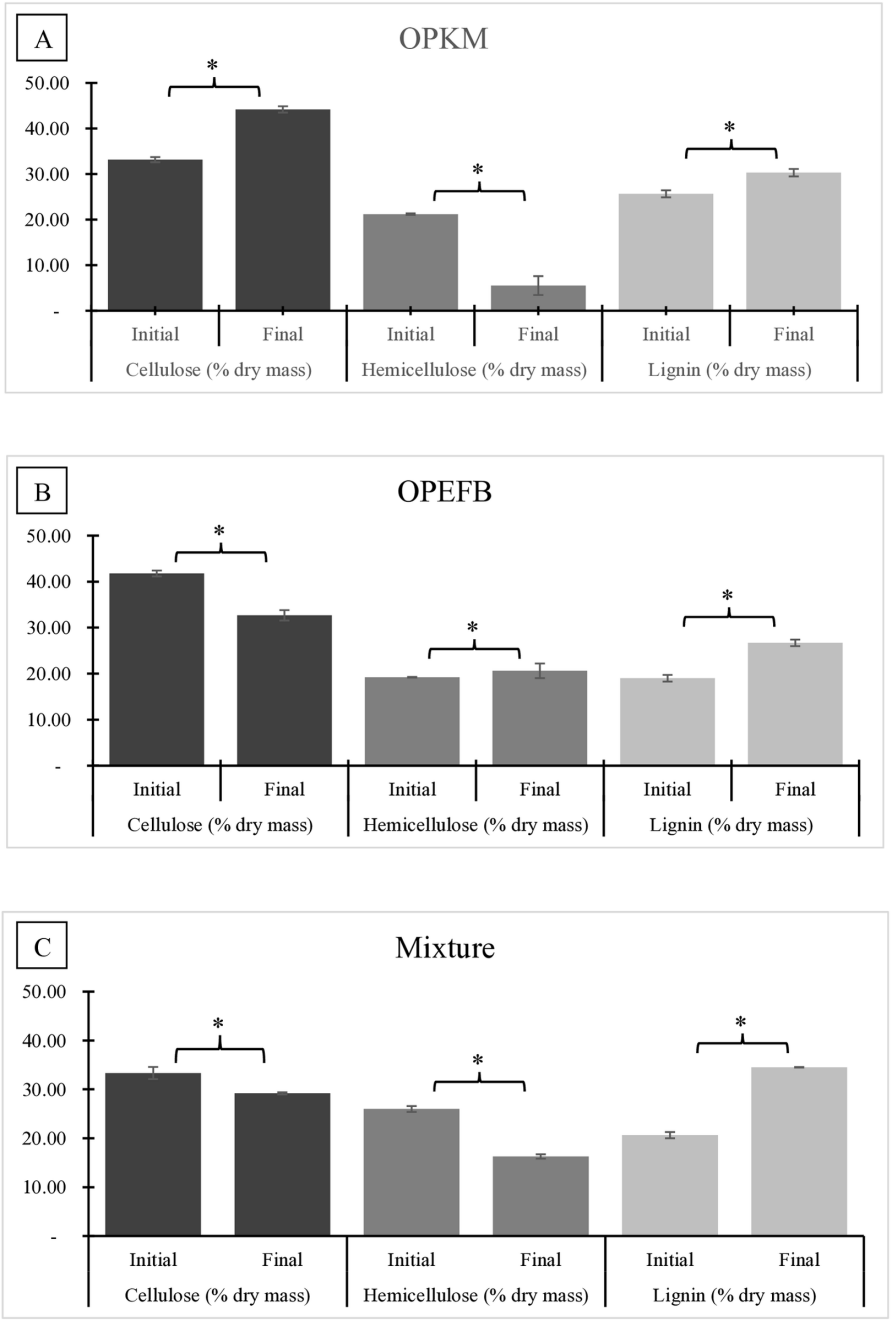
Lignocellulose composition changes of (A) OPKM, (B) OPEFB, and (C) the mixed substrate after being fed to BSFL.

Fig 1 illustrates the dynamics of cellulose, hemicellulose, and lignin in OPKM, OPEFB, and the mixed substrate during digestion by BSFL.

The most significant cellulose decrease occurred in OPEFB at 21.78 ± 1.13%, from 41.78 ± 0.64% to 32.68 ± 1.13% (Fig 1B). However, in OPKM substrate, the cellulose content percentage tends to increase by 33.27 ± 0.69%, due to its being concentrated caused by depletion of other physicochemical characters. The hemicellulose content of OPKM significantly decreased by 71.37 ± 2.07%, from 21,18 ± 0,19% to 5,51 ± 2,07% (Fig 1A). While in the OPEFB substrate, as other physicochemical characteristics are depleted, the hemicellulose content percentage tends to increase by 4.95 ± 1.59%. The decreasing of cellulose and hemicellulose occurred in the co-digestion mixed substrate; it was 12.47 ± 0.22% and 26.18 ± 0.45%, respectively (Fig 1C). The reduction in lignin across the three substrate types did not indicate any decrease in value, reflecting the lack of BSFL ability to digest the lignin contained in these substrates.

By-products of oil palm, including OPKM and OPEFB, are organic materials with a high lignocellulose content [14]. Yang et al. [6] explained that BSFL has a various gut microbial community (including fungi and bacteria) that significantly influenced by dietary intake. Fungi and bacteria are microorganisms recognized for their production of lignocellulose-degrading enzymes, including endoglucanases, exoglucanases, and β-glucosidases for cellulose degradation, as well as endo-1,4-β-xylanase, β-xylosidase, β-mannanases, β-glucosidases, and hemicellulolytic esterases for hemicellulose degradation, into simpler sugars [25,26]. BSFL utilizes these simple sugars as a source of nutrition and for producing the body biomass during the bioconversion process [27].

#### Oil Palm Kernel Meal (OPKM).

The C/N ratio in BSFL growing media of OPKM is 17.24 ± 0.02, which is within the acceptable range of 15–30 for fungi growth [28]. According to experiment Klüber et al. [8] a large number of fungi was discovered in the gut of BSFL which consumed OPKM. In the study, 75% of the fungi identified belonged to the phylum Ascomycota, with the dominant families were Saccharomycetaceae, Pichiaceae, Debaryomycetaceae, and Nectriaceae.

In a study conducted by Zhou et al. [29], *Kluyveromyces marxianus*, derived from the *Kluyveromyces* genus of the Saccharomycetaceae family, is recognized for its ability to produce hemicellulase enzymes, specifically xylanase and mannanase, reaching 25,600 U/mL and 10,200 U/mL, respectively. High xylanase production activity was observed during experiments using the *Pichia kudriavzevii* strain from the Pichiaceae family [30]. The production of hemicellulose enzymes was observed as well in a strain of *Candida tropicalis* from Debaryomycetaceae family [30], including the strains of *Fusarium commune* from Nectriaceae family [31]. This may explain the significant degradation of hemicellulose content in OPKM substrates consumed by BSFL.

#### Oil Palm Empty Fruit Bunch (OPEFB).

On the other hand, the degradation of cellulose in the OPEFB substrate was more significant than hemicellulose. As previously discussed, OPEFB is recognized as an organic material that is low in nutrients and thus induce starvation of BSFL. According to experiments conducted by Yang et al. [6], under starvation conditions, one of the most abundant BSFL gut microbes that develops were the Actinobacteria phylum with the most genus specifications being *Actinomyces, Microbacterium, Leucobacter,* and *Sphingobacterium*. Microbes of the Actinobacteria phylum are recognized for their ability to convert cellulose into simple glucose units by the synthesis of abundant cellulolytic enzymes (cellulases), compared to enzymes that degrade hemicellulose [32,33]. Therefore, this microbial activity causes the degradation of cellulose to be more aggressive than hemicellulose in the OPEFB substrate.

#### Co-digestion mixture.

In co-digestion mixture treatment, both cellulose and hemicellulose decreased, possibly due to several factors that ultimately improve and enhance the quality of the substrate. The beneficial gut bacteria generated by BSFL during the conversion process facilitates the BSFL as hosts in digestion, absorption, and metabolism of complex nutrients [13]. Proteins in OPKM may function as substrates for microbial activity during bioconversion process. Gibson et al. [34] explained that the bacteria utilize proteins as a nutritional source for growth and reproduction. Proteins are broken down into amino acids by enzymatic activity, which are subsequently utilized for the synthesis of cellular components, including enzymes, membranes, and other structures. Moreover, bacteria can synthesize proteins as a component of their metabolism or biomass [24,34]. Furthermore, in the co-digestion treatment, the C/N ratio was at an ideal condition (23.33 ± 0.33) for fungi growth [28].

The presence of these bacteria and fungi positively influences the degradation of cellulose and hemicellulose in the substrate, due to the cellulase and hemicellulase enzymes synthesized by these microorganisms. This information is further supported by the investigations from Klüber et al. [14], which indicate that in BSFL fed with OPKM and OPEFB, the proliferation of fungi and bacteria that produce cellulase and hemicellulase enzymes was observed in the gut of BSFL. This investigation revealed that fungi were predominantly from the Ascomycota phylum, primarily represented by the genera Trichocomaceae, Aspergillaceae, and Plectosphaerellaceae, as well as the Basidiomycota phylum, which was mostly identified by the genus Trichosporonaceae. Concurrently, regarding the growth of bacteria observed in the gut of BSFL fed with OPKM and OPEFB, the Bacillota phylum (previously named Firmicutes) is predominant, with the most genera from Bacillaceae, Lachnospiraceae, Paenibacillaceae, Enterococcaceae, Planococcaceae, and Ruminococcaceae. This is followed by the Pseudomonadota phylum (previously named Proteobacteria), which contains the most genera from Enterobacteriaceae, Xanthomonadaceae, Rhizobiaceae, Pseudomonadaceae, Caulobacteriaceae, and Burkholderiaceae. Furthermore, the Bacteroidota phylum is represented by the most genera from Sphingobacteriaceae, Chitinophagaceae, and Flavobacteriaceae [14].

Haq et al. [35] explained that cellulose is a crystalline structure formed by densely arranged glucose monomers interconnected by β-1,4 glycosidic linkages, with a high number of hydroxyl groups in its lateral fibers promoting hydrogen bonds, making it stable and resistant to depolymerization. Meanwhile hemicellulose is a branching polysaccharide that has amorphous characteristics and contains a lower degree of polymerization. This characteristic causes it more susceptible to degradation than cellulose. Therefore, in this case, the microbial activity is more aggressive to hemicellulose than to cellulose.

### Morphology changes of substrates

**Fig 2:**
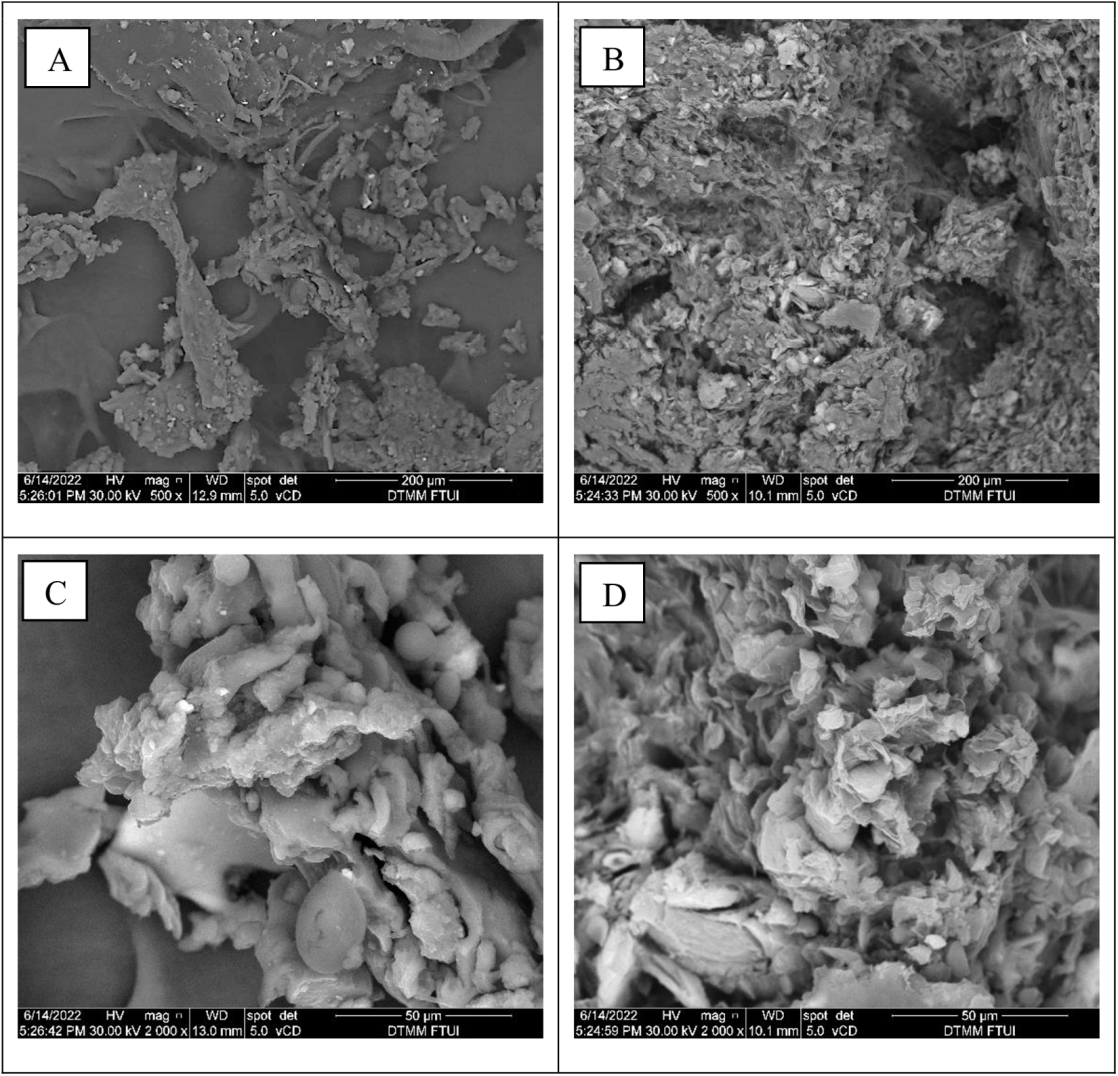
Scanning electron microscope micrographs. (A) OPKM substrate before digestion (500 × magnification); (B) OPKM substrate after digestion (500 × magnification); (C) OPKM substrate before digestion (2000 × magnification); (D) OPKM substrate after digestion (2000 × magnification) with black soldier fly larvae.

Fig 2 displays the substrate morphology of OPKM as analyzed by scanning electron microscope (SEM) before and after to BSFL treatment, using two magnification levels (500× and 2000×).

Before digestion, the surface of OPKM is organized densely and systematically in big aggregates (Fig 2A and 2C). Meanwhile, in the SEM results post-digestion, the surface morphology of OPKM appears dispersed and fractured (Fig 2B and 2D). Microstructural changes and deterioration of OPKM may result from the feeding activity of BSFL, assisted by extracellular enzymes. Fiber-degrading enzymes are synthesized by several fungi and bacteria, which are excreted by BSFL during the bioconversion process [8], leading to the decomposition of fibers and other structural constituents of OPKM [36]. Therefore, the decomposition enhances the contact area between microbial enzymes and OPKM, thereby promoting the release of bioactive substances that can be digested by BSFL [37].

**Fig 3:**
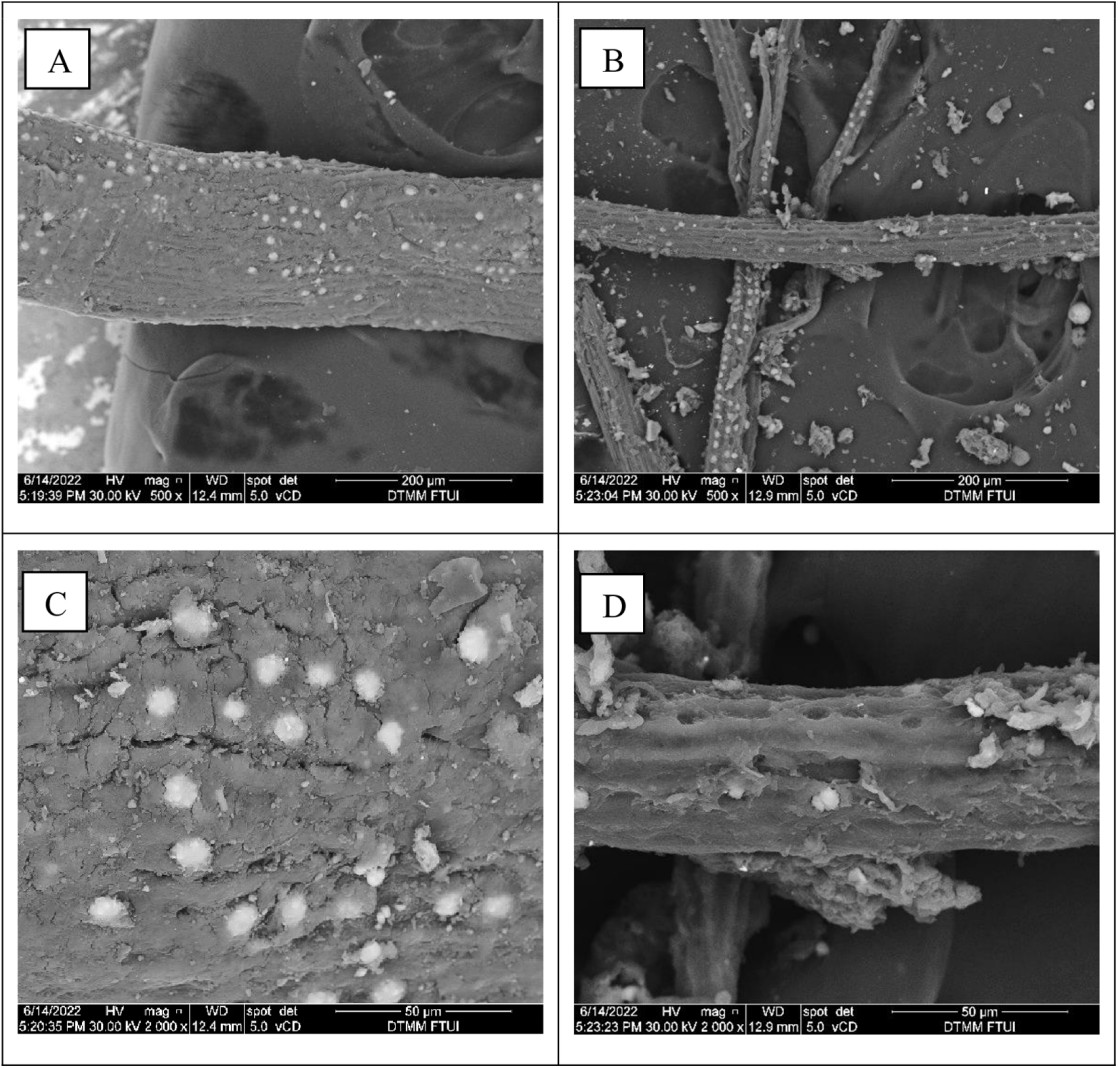
Scanning electron microscope micrographs. (A) OPEFB substrate before digestion (500 × magnification); (B) OPEFB substrate after digestion (500 × magnification); (C) OPEFB substrate before digestion (2000 × magnification); (D) OPEFB substrate after digestion (2000 × magnification) with black soldier fly larvae.

SEM analysis was also conducted to observe the changes in the substrate morphology of OPEFB with two magnifications (500× and 2000×), as shown in Fig 3. On the surface of OPEFB before digestion, it revealed solid, dense, and compact (Fig 3A and 3C). Meanwhile, in the SEM results after digestion, the surface morphology of OPEFB shows cavities, cracks, and pores (Fig 3B and 3D). OPEFB deterioration and structural changes may result from BSFL feeding activity, which is aided by extracellular enzymes. As explained by [6], various microbes derived from the secreted BSFL gut can produce enzymes capable of degrading cellulose and hemicellulose during the bioconversion process. The SEM analysis of OPEFB treated with vermicompost showed similar results, revealing a rough and broken surface with holes, creating a larger surface area that allows for increased surface adhesion for microbes in the earthworm gut and microflora, which causes further degradation [38]. The white region observed in the SEM analysis is a number of Silica crystallite bodies bound between the fibers [39].

**Fig 4:**
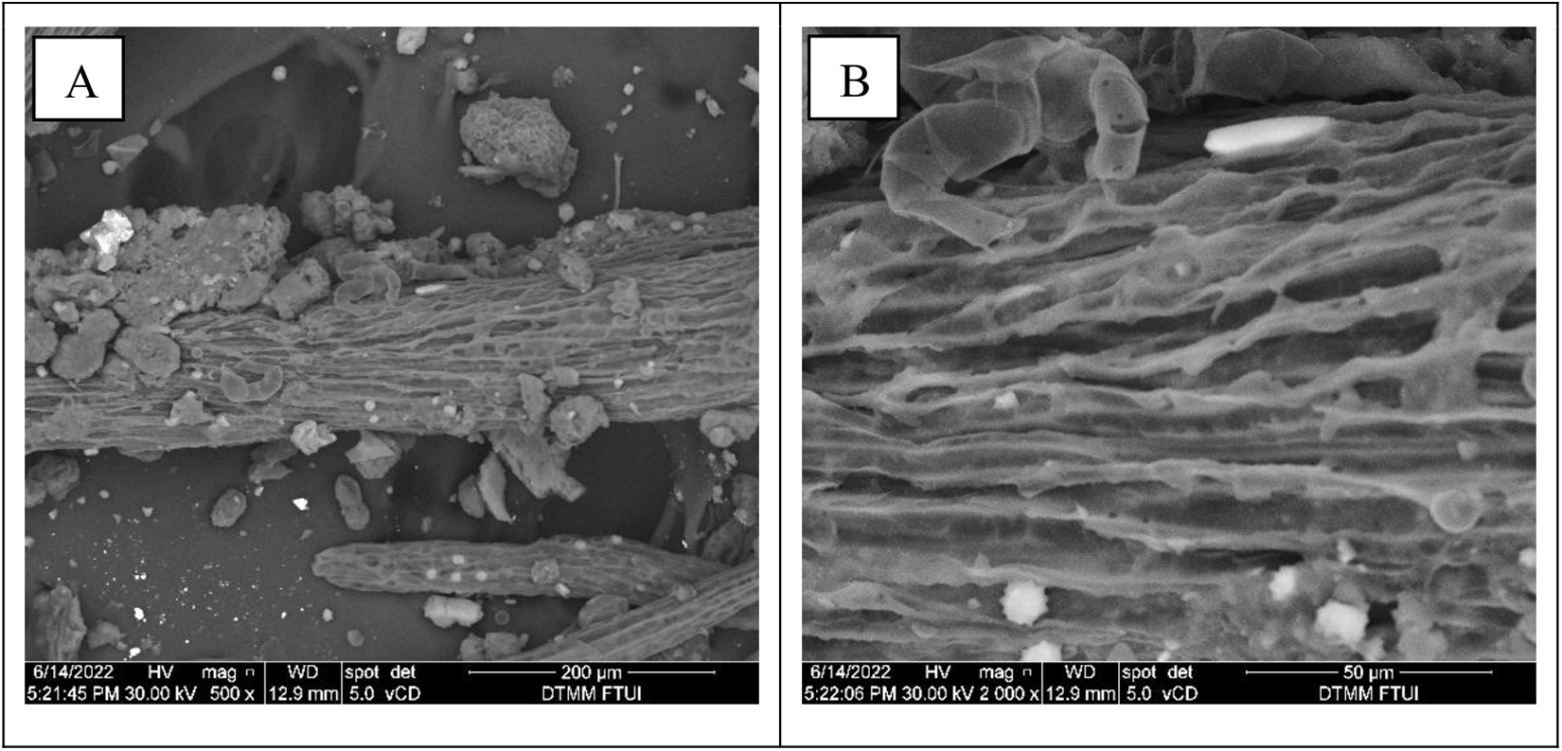
Scan electron microscope micrographs. (A) co-digestion mixtures substrate after digestion (500 × magnification); (B) co-digestion mixtures substrate after digestion (2000 × magnification) with black soldier fly larvae.

The SEM analysis results of the co-digestion mixtures substrate clearly demonstrate the changes in substrate morphology post-digestion by BSFL at both 500× and 2000 × magnification (Fig 4). The surface of the co-digestion mixtures substrate shows deterioration, fragmentation (Fig 4A), hollows, fissures, and more pores (Fig 4B). Microstructural alterations and damage to co-digestion mixtures substrate may also result from the feeding behavior of BSFL, which is facilitated by the activities of gut BSFL bacteria, as detailed by Klüber et al. [14]. The co-digestion approach is appropriate for application in BSFL production for dealing with high lignocellulosic organic material, such as oil palm by-products, OPKM and OPEFB. This discovery aligns with the experiment conducted by Rehman et al. [12], which discovered that BSF larvae cultivated on a co-digestion of dairy and chicken manure had a more porous and corrugated surface after digestion, but a relatively compact and rigid surface prior to BSFL degradation.

## Conclusion

This study has shown that black soldier flies have a significant ability to degrade high lignocellulosic organic materials, such as oil palm by-products (OPKM and OPEFB) and then convert them into high-value products. However, due to its low nutritional content, OPEFB is one of the organic materials that BSFL finds extremely difficult and sluggish to digest directly. By adding OPKM, the co-digestion method could improve the digestibility of OPEFB as BSFL feed. A significant difference occurred in the treatment of BSFL fed with co-digestion mixture substrate compared to BSFL fed with solely OPEFB. This improvement is reflected in changes in the substrate that were assessed visually through SEM and physicochemical analysis. The survival rate of BSFL fed with co-digestion mixed substrates increased to 77.63%, producing a final fresh weight of 78.5 mg per larvae in twenty-five days, with the feed conversion ratio reduced to 5.74. Furthermore, the degradation of oil palm biomass by BSF achieved a higher waste reduction index of 76.57%, accompanied by a bioconversion efficiency of 5.18%. Ultimately, the current finding shows the potential of BSFL in the utilization of organic materials with high lignocellulosic content, such as oil palm by-products. Future research could focus on the optimization of the mixing ratio of OPKM and OPEFB, or other potential substrates, to attain an FCR value nearest that of pure OPKM (2.13). Following this, it would be beneficial to implement larger-scale applications to enhance the processing of organic materials and yield more cost-effective products. This approach is promising for implementation in palm oil processing plants as an environmentally sustainable strategy to manage waste or by-products generated.

## Supporting information

S1 File Raw data for Table 1. (PDF)

S2 File Raw data for Table 2. (PDF)

S3 File Raw data for Table 3. (PDF)

S4 File Raw data for Fig 1. (PDF)
