# Supplementary material for: Substrate degradation and black soldier fly larvae bioconversion performance profile on co-digested oil palm biomass-based feedstock
Source: PLoS One. 2025 Sep 15;20(9):e0332046. doi: 10.1371/journal.pone.0332046 (PMC12435691; doi:10.1371/journal.pone.0332046)
Supplement: S1 File — (PDF) [file pone.0332046.s001.pdf]

Raw data for Table 1 Physicochemical characteristics of the OPKM, OPEFB, and the mixed substrates

Parameters	OPKM	OPEFB	Mixed substrate
Water Content (%)	7.88	4.66	6.27
	7.89	4.65	6.28
	7.86	4.63	6.25
Dry Mass (%)	92.12	95.34	93.73
	92.11	95.35	93.72
	92.14	95.37	93.75
Protein (%)	20.01	3.65	14.52
	19.95	3.72	14.14
	19.98	3.69	14.33
Lipid (%)	5.78	0.32	2.32
	4.56	0.46	1.39
	5.17	0.39	1.86
Cellulose (% dry mass)	32.58	42.43	32.10
	33.74	41.14	34.60
	33.15	41.79	33.36
Hemicellulose (% dry mass)	21.00	19.29	26.60
	21.38	19.12	25.39
	21.19	19.21	25.99
Lignin (% dry mass)	26.42	18.28	21.30
	24.88	19.74	20.00
	25.66	19.00	20.65
Total Organic Carbon (% dry mass)	55.13	49.51	53.34
	54.99	49.59	53.39
	55.20	49.99	53.74
Total Nitrogen (% dry mass)	3.20	1.43	2.32
	3.19	1.40	2.26
	3.20	1.42	2.29
C/N ratio	17.22	34.63	22.95
	17.24	35.42	23.60
	17.27	35.33	23.44

MEAN

Parameters	OPKM	OPEFB	Mixed substrate
Water Content (%)	7.88	4.65	6.27
Dry Mass (%)	92.12	95.35	93.73
Protein (%)	19.98	3.68	14.33
Lipid (%)	5.17	0.39	1.85
Cellulose (% dry mass)	33.15	41.78	33.35
Hemicellulose (% dry mass)	21.18	19.20	25.99
Lignin (% dry mass)	25.65	19.01	20.64
Total Organic Carbon (% dry mass)	55.11	49.69	53.49
Total Nitrogen (% dry mass)	3.19	1.41	2.29
C/N ratio	17.24	35.12	23.33

STDEV

Parameters	OPKM	OPEFB	Mixed substrate
Water Content (%)	0.02	0.02	0.02
Dry Mass (%)	0.02	0.02	0.02
Protein (%)	0.02	0.03	0.19
Lipid (%)	0.61	0.06	0.46
Cellulose (% dry mass)	0.57	0.64	1.24
Hemicellulose (% dry mass)	0.19	0.08	0.60
Lignin (% dry mass)	0.77	0.73	0.64
Total Organic Carbon (% dry mass)	0.10	0.25	0.21
Total Nitrogen (% dry mass)	0.01	0.01	0.03
C/N ratio	0.02	0.43	0.33
