# Supplementary material for: Substrate degradation and black soldier fly larvae bioconversion performance profile on co-digested oil palm biomass-based feedstock
Source: PLoS One. 2025 Sep 15;20(9):e0332046. doi: 10.1371/journal.pone.0332046 (PMC12435691; doi:10.1371/journal.pone.0332046)
Supplement: S2 File — (PDF) [file pone.0332046.s002.pdf]

Raw data for Table 2 Survival rate, development time, and larval production of BSFL reared on OPKM, OPEFB and the mixed substrate

Feeding Treatment	Survival Rate (%)	Development time (days)	Fresh Weight		Individual larval length (mm)
			Total Larval (g)	Individual Larval (mg)	
OPKM	86.14	20.1	1,191.40	208.08	21.00
	86.19	20.0	1,192.16	199.52	22.00
	86.17	20.0	1,191.78	203.80	21.50
OPEFB	67.07	40.1	69.54	10.88	9.00
	66.76	40.0	69.27	10.53	9.00
	66.91	40.0	69.40	10.70	8.00
Mixed substrate	77.74	25.0	442.78	78.71	17.00
	77.75	25.0	442.87	78.31	16.90
	77.41	25.0	440.92	78.51	16.95

MEAN

Feeding Treatment	Survival Rate (%)	Development time (days)	Fresh Weight		Individual larval length (mm)
			Total Larval (g)	Individual Larval (mg)	
OPKM	86.16	20	1,191.78	203.79	21.50
OPEFB	66.91	40	69.40	10.70	8.66
Mixed substrate	77.63	25	442.19	78.50	16.95

STDEV

Feeding Treatment	Survival Rate (%)	Development time (days)	Fresh Weight		Individual larval length (mm)
			Total Larval (g)	Individual Larval (mg)	
OPKM	0.02	0.02	0.37	4.27	0.50
OPEFB	0.15	0.02	0.13	0.17	0.57
Mixed substrate	0.19	0.02	1.09	0.19	0.05
