# Supplementary material for: Substrate degradation and black soldier fly larvae bioconversion performance profile on co-digested oil palm biomass-based feedstock
Source: PLoS One. 2025 Sep 15;20(9):e0332046. doi: 10.1371/journal.pone.0332046 (PMC12435691; doi:10.1371/journal.pone.0332046)
Supplement: S3 File — (PDF) [file pone.0332046.s003.pdf]

Raw data for Table 3 Waste reduction, bioconversion efficiency, and feed conversion ratio of BSFL converting OPKM, OPEFB and the mixed substrate

Feeding Treatment	Waste Reduction (%)	Bioconversion Efficiency (%)	Feed Conversion Ratio
OPKM	81.34	16.81	2.13
	79.82	16.82	2.13
	79.87	16.81	2.13
OPEFB	57.69	1.04	36.65
	55.46	1.04	36.79
	55.91	1.04	36.72
Mixed substrate	75.96	5.19	5.74
	77.35	5.20	5.74
	76.41	5.17	5.77

MEAN

Feeding Treatment	Waste Reduction (%)	Bioconversion Efficiency (%)	Feed Conversion Ratio
OPKM	80.34	16.81	2.13
OPEFB	56.35	1.03	36.72
Mixed substrate	76.57	5.18	5.74

STDEV

Feeding Treatment	Waste Reduction (%)	Bioconversion Efficiency (%)	Feed Conversion Ratio
OPKM	0.86	0.01	0.01
OPEFB	1.17	0.01	0.07
Mixed substrate	0.70	0.01	0.01
