# Supplementary material for: Substrate degradation and black soldier fly larvae bioconversion performance profile on co-digested oil palm biomass-based feedstock
Source: PLoS One. 2025 Sep 15;20(9):e0332046. doi: 10.1371/journal.pone.0332046 (PMC12435691; doi:10.1371/journal.pone.0332046)
Supplement: S4 File — (PDF) [file pone.0332046.s004.pdf]

Raw data for Figure 1 Lignocellulose composition changes of (A) OPKM, (B) OPEFB, and (C) the mixed substrate after being fed to BSFL.

Parameters		OPKM	OPEFB	Mixed substrate
Cellulose (% dry mass)	Initial	32.58	42.43	32.10
		33.74	41.14	34.60
		33.15	41.79	33.36
	Final	44.88	31.56	29.43
		43.49	33.83	28.97
		44.19	32.66	29.20
Hemicellulose (% dry mass)	Initial	21.00	19.29	26.60
		21.38	19.12	25.39
		21.19	19.21	25.99
	Final	4.00	21.04	16.12
		7.04	20.20	16.43
		5.51	20.64	16.28
Lignin (% dry mass)	Initial	26.42	18.28	21.30
		24.88	19.74	20.00
		25.66	19.00	20.65
	Final	31.12	27.40	34.45
		29.47	25.97	34.60
		30.30	26.71	34.53

MEAN

Parameters		OPKM	OPEFB	Mixed substrate
Cellulose (% dry mass)	Initial	33.15	41.78	33.35
	Final	44.18	32.68	29.19
Hemicellulose (% dry mass)	Initial	21.18	19.20	25.99
	Final	5.51	20.62	16.27
Lignin (% dry mass)	Initial	25.65	19.00	20.64
	Final	30.29	26.69	34.52

STDEV

Parameters		OPKM	OPEFB	Mixed substrate
Cellulose (% dry mass)	Initial	0.57	0.64	1.24
	Final	0.69	1.13	0.22
Hemicellulose (% dry mass)	Initial	0.19	0.08	0.60
	Final	2.07	1.59	0.45
Lignin (% dry mass)	Initial	0.77	0.73	0.64
	Final	0.82	0.71	0.07
